# Drivers of rhizosphere bacterial communities in *Atriplex canescens* across soil depth and growth stage in an extra arid region

**DOI:** 10.3389/fmicb.2026.1743201

**Published:** 2026-06-29

**Authors:** Fei Chen, Hailian Liang, Wenjie Zhou, You Wang, Ruiheng Lyu

**Affiliations:** 1 College of Horticulture and Forestry, Tarim University, Alar, China; 2 Farmland Shelterbelt Ecosystem Observation and Research Station, Tarim Basin, Alar, China; 3 College of Life Sciences, Tarim University, Alar, China; 4 Ocean University of China, Qingdao, China

**Keywords:** *Atriplex canescens*, driving mechanism, extreme arid region, rhizosphere soil bacterial community, spatiotemporal variation

## Abstract

In the restoration of extreme arid desert ecosystems, plant-rhizosphere microorganism-soil synergy determines vegetation colonization and stability. This study focused on *Atriplex canescens*, divided soil into 0–20, 20–30, 30–40 cm layers, and explored spatiotemporal dynamics of its rhizosphere bacterial community and driving mechanisms. Results showed: ① Dominant phyla were *Pseudomonadota*, *Actinomycetota*, *Bacillota*, *Bacteroidota* (cumulative relative abundance >70%); *Ralstonia* was enriched in early growth period (EP), while *Halomonas* and *Arthrobacter* dominated middle (MP) and late (LP) periods. ② Significant spatiotemporal variation existed: temporally, *α*-diversity, network complexity and stability in MP/LP were higher than EP; spatially, bacteria enriched in root-intensive layers (20–30, 30–40 cm), with a “suitable microhabitat” in 20–30 cm layer during EP. ③ Growth period and soil depth synergistically regulated the community by altering spatiotemporal heterogeneity of soil nitrogen, dissolved organic carbon and pH, and nitrogen and dissolved organic carbon were core factors in root-intensive layers, while pH played a greater role in surface and deep layers. ④ The core regulatory chain: growth period and soil depth formed a two-dimensional framework, modifying root-intensive layer microenvironment to induce soil physicochemical heterogeneity, driving bacterial community differentiation and forming patterns adapted to *Atriplex canescens* at different growth stages. This study clarified the community’s spatiotemporal dynamics and driving mechanisms, providing theoretical support for microecological regulation in desert restoration.

## 1 Preface

As the core driver of material cycling and energy flow in terrestrial ecosystems (Wang et al., 2024), the rhizosphere bacterial community indirectly maintains the structural stability and functional balance of ecosystems by participating in key processes such as soil remediation (Li et al., 2020) and nutrient transformation regulation (Hao et al., 2023), serving as a crucial link connecting plants and the soil environment (Siegieda et al., 2024; Bachar et al., 2009; Bastian et al., 2009). Among them, beneficial microorganisms such as *Azotobacter* spp. and *Phosphobacteria* spp. can significantly improve soil nutrient availability, directly supporting plant growth and development; in contrast, pathogenic bacteria may interfere with vegetation restoration through the release of metabolic toxins (Mendes et al., 2013; Romero et al., 2012). Therefore, the structural characteristics of the rhizosphere bacterial community are not only a core indicator of soil health (Yang X. M et al., 2024), but also their adaptability to plant growth needs is a key factor determining the effectiveness of ecological restoration. Dynamic changes in environmental factors such as soil pH, nutrient content, and water content directly drive the composition and structure of the rhizosphere bacterial community (Kumar et al., 2021; Shcherbakova et al., 2017); as the “second genome” of plants, rhizosphere bacteria form complex interaction networks through material, energy, and information exchange (Hao et al., 2023), encompassing diverse relationships such as predation, competition, and mutualism (Gutknecht et al., 2012; Zhu et al., 2020; Kumar et al., 2021). The stability of this network structure directly affects community function and environmental adaptability (Rooney et al., 2006; Montoya et al., 2006).

Against the backdrop of global climate change, extreme arid regions are facing intensified coupled stress from multiple factors including drought, high temperature, and salinity (Shi et al., 2022; Liu et al., 2019; Sheik et al., 2011; Oliverio et al., 2017; Cordero et al., 2023). The asymmetric reduction in precipitation frequency and intensity has further amplified the vulnerability of the rhizosphere microecosystem (Barnard et al., 2013). In this context, deciphering the spatiotemporal variation patterns and their driving factors of the rhizosphere bacterial community is of great theoretical and practical value for optimizing ecological restoration strategies in extreme arid regions and enhancing ecosystem resilience (Bandopadhyay et al., 2024; Zhang et al., 2012).

*Atriplex canescens* (*A. canescens*) is a perennial semi-evergreen halophytic shrub belonging to the genus *Atriplex* in the amaranth family. Native to the arid, semi-arid, and saline-alkali desert regions of western North America, it serves as an excellent pioneer tree species for ameliorating saline-alkali soils, restoring degraded grasslands, and controlling soil erosion. Its branches and leaves are also an important woody forage resource, while the species is widely used in ecological engineering projects such as roadside greening and windbreak and sand fixation in arid regions. This plant exhibits strong tolerance to drought, salinity, and poverty (Ma et al., 2022). Its unique physiological metabolic mechanisms (such as salt bladder structures and succulent leaves) enable it to grow stably under combined drought, high temperature, salinity stress. Meanwhile, it regulates the rhizosphere microenvironment through root exudates to provide specific living conditions for rhizosphere bacteria (Riley and Barber, 1971), playing an irreplaceable role in windbreak and sand fixation, soil improvement, and ecological restoration in extreme arid regions. Vegetation restoration practices in extreme arid regions have shown that the ecological adaptability of *A. canescens* is closely related to its rhizosphere microecosystem. However, there is still a lack of systematic understanding of the spatiotemporal dynamic characteristics of its rhizosphere bacterial community, the adaptation rules to plant growth needs, and the regulatory mechanisms. As a dominant species for ecological restoration in extreme arid regions, using *A. canescens* as a research carrier to decipher the regulatory mechanisms of halophytes on the rhizosphere bacterial community not only clarifies the interaction strategy between the plant and rhizosphere bacteria but also provides scientific references for the cultivation and management of similar halophytes and the improvement of ecological restoration efficiency, highlighting the object specificity and practical necessity of this study (Baker et al., 2024; Pratiwi et al., 2024).

In recent years, research on plant-rhizosphere bacteria interactions in saline-alkali environments has made certain progress: Studies have shown that rhizospheric bacteria from halophytes exhibit unique community dynamics and plant growth-promoting traits (Castro-Severyn et al., 2024). Jones et al. (2023) demonstrated that plant roots create nutrient cycling hotspots in the rhizosphere, sustaining microbial activity under extreme conditions. Drought-tolerant legumes recruit *Bacillus* as a dominant plant growth-promoting genus in the rhizosphere, root endosphere, and seed niches (Mataranyika et al., 2024; Liu et al., 2025). The halophyte *Halocnemum strobilaceum* increases rhizosphere bacterial population size and diversity by up to 100-fold compared to bulk soil, with specific affinity for the genus *Halomonas* (Behairi et al., 2022; Cao et al., 2022; Dai et al., 2018; Liu et al., 2025). Liu B. S. et al. (2022) found significant differences in the rhizosphere bacterial community of *Leymus chinensis* under different saline-alkali gradients; studies have confirmed that plants in saline-alkali soil can recruit high-abundance keystone bacteria through rhizosphere effects to form complex co-occurrence networks (Liu et al., 2023; Li et al., 2024a,b); research on *Citrus reticulata* in coastal saline-alkali land showed that rhizosphere bacteria help plants resist iron deficiency stress (Jiang et al., 2024); studies on the diversity, composition, and functions of endophytic bacteria in *Medicago sativa* roots under saline-alkali stress have screened out key strains related to salt-alkali tolerance (Yang D. H. et al., 2024), and inoculation with plant growth-promoting rhizobacteria and rhizobia significantly improved alfalfa resistance to abiotic stress (Sun et al., 2025). Liu B. S. et al. (2022) found that there are significant differences in the rhizosphere bacterial community of *Leymus chinensis* under different saline-alkali gradients; relevant studies have confirmed that plants in saline-alkali soil can recruit high-abundance key bacteria through rhizosphere effects to form complex bacterial network structures (Liu et al., 2023). research on *Citrus reticulata* in coastal saline-alkali land has shown that rhizosphere bacteria can help plants resist iron deficiency stress (Jiang et al., 2024). studies on the diversity, composition, and functions of endophytic bacteria in the roots of *Medicago sativa* under saline-alkali stress have screened out key strains related to salt-alkali tolerance (Yang D. H. et al., 2024), and inoculation with plant growth-promoting rhizobacteria and rhizobia can significantly improve the resistance of alfalfa to abiotic stress (Sun et al., 2025; Li et al., 2025). However, existing studies still have three core gaps, which seriously restrict the in-depth understanding of plant-rhizosphere bacteria interaction mechanisms in extreme arid regions: first, most focus on a single stress factor (such as individual salinity-alkali or drought), ignoring the real habitat characteristics of coupled drought-high temperature-salinity factors in extreme arid regions, resulting in research conclusions that are difficult to directly apply to actual ecological restoration scenarios; second, most separately analyze the single impact of growth period or soil environment, lacking systematic research under the dual-variable coupling of “growth period × soil depth”, making it impossible to reveal the spatiotemporal regulation rules of plants on the rhizosphere microbial community; third, the mechanism by which halophytes actively regulate the structure and adaptability of the rhizosphere bacterial community is still unclear, especially the lack of comprehensive analysis based on the associations between community diversity, composition structure, co-occurrence network, and soil physicochemical properties, making it difficult to clarify the interaction path of “plant-microbe-soil.” These research gaps provide clear innovative entry points for this study and highlight the urgency and necessity of conducting this research.

Based on this, this study takes *A. canescens* of the same stand age in a typical extreme arid region of the Tarim Basin, China, as the research object, focusing on the spatiotemporal dimension of growth period and soil depth, systematically explores the variation rules of diversity, composition structure, and co-occurrence network of the rhizosphere bacterial community, and clarifies the mediating role of soil physicochemical properties in the regulation of rhizosphere bacterial community adaptability by *A. canescens* (Layeghtifard et al., 2017). Accordingly, the following two research hypotheses are proposed: (1) *A. canescens* drives significant spatiotemporal differences in the diversity, composition structure, and co-occurrence network interaction patterns of the rhizosphere bacterial community through growth period regulation and adaptation to soil microenvironment, and these differences reflect adaptive characteristics to plant growth needs; (2) Soil physicochemical properties are the key factors mediating the spatiotemporal regulation of rhizosphere bacterial community structure and adaptability by *A. canescens*, and the dominant regulating physicochemical factors vary in different growth periods or soil depths.

## 2 Materials and methods

### 2.1 Overview of the research landscape

**Figure 1:**
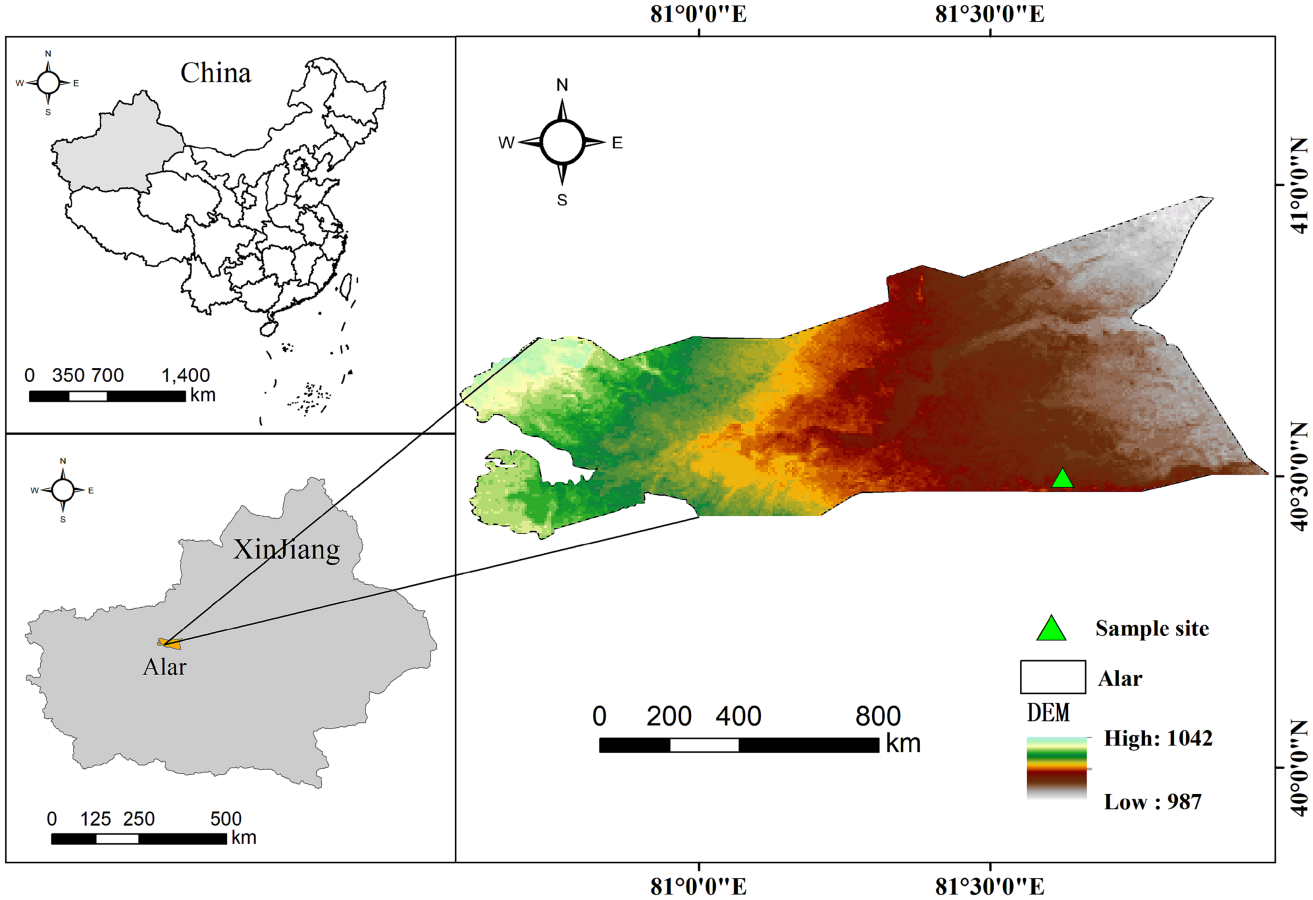
Overview map of the study area.

The study area is located in the Eleventh Regiment of Alar City, the First Division of Xinjiang Production and Construction Corps, China (Figure 1), which is situated in a sandy and windy area of the Taklamakan Desert, with a temperate continental climate and an average elevation of 1,011 m. The annual precipitation in 2023 was 68 mm. The average annual temperature was 10.7 °C, and the annual evaporation was 2,500 mm. The soil texture in this area is sandy, and the main artificially planted vegetation in the region is *A. canescens*, with associated vegetation including *Tamarix ramosissima*, *Alhagi sparsifolia Shap*, *Launaea polydichotoma*, and *Karelinia caspia*.

### 2.2 Experimental method

A *A. canescens* windbreak and sand-fixation forest, established in 2018, served as the artificial vegetation. It was planted with a 2.0 m row spacing, under drip irrigation, and fertilized only with base fertilizer at establishment. Rhizosphere soil samples were collected in May, August, and November 2023. The day of sample collection was sunny and cloudless with no rainfall for 7 consecutive days. Based on phenological characteristics, the growing season of this species is divided into three stages: the early growth periods (EP; April–June), the middle growth periods (MP; July–August), and the late growth periods (LP; September–November). In the artificial cultivation area of *A. canescens*, a flat area with 100 × 100 m terrain was selected as a sample plot, and all *A. canescens* individuals in the sample plot were examined, and sample trees were selected based on plant height, crown diameter, and ground diameter. Subsequently, nine standard trees were randomly selected, and their rhizosphere soils were collected. The morphological characteristics of the standard tree are as follows: plant height is 74.47 ± 14.39 cm, crown diameter is 54.47 ± 9.55 cm (north–south) × 50.73 ± 11.44 cm (east–west), and ground diameter is 3.42 ± 0.85 cm. Based on the field survey records of habitat conditions for the *A. canescens* community, soil physicochemical analysis was further conducted to quantify its soil environmental characteristics.

#### 2.2.1 Rhizosphere soil sample collection

Rhizosphere soil samples were collected as follows: four 1 × 1 × 1 m soil profiles were collected in the east, south, west, and north directions at a distance of 0.5 m from the basal diameter of the *A. canescens* sample tree, and soil samples were collected from soil depths of 0–20, 20–30, and 30–40 cm (diameter of the auger = 10 cm) using soil auger method after the removal of apoplectic material on the surface. The basis for this depth division design is that previous studies have clearly confirmed that the roots of *A. canescens* are concentrated in the 20–40 cm soil layer; therefore, this root-dense layer was further subdivided for sampling to more accurately capture the differences in soil characteristics within this key soil layer. Fine roots (≤2 mm) were selected from the soil with sterile forceps at a low temperature (4 °C), and loose soil was removed by shaking the root system according to the shaking method of Riley and Barber (1971), retaining the soil tightly adhered to the root surface (approximately 1 mm in thickness as visually estimated) as rhizosphere soil. Subsequently, the soil attached to the roots was gently brushed off with a sterile brush using the root brushing method, and this soil was considered as the rhizosphere soil sample. The rhizosphere soil from the same four directions of the same soil depth of the same *A. canescens* plants was mixed as one sample and considered one replicate. A total of six healthy plants were selected from the nine initially sampled individuals (as described in Section 2.2) based on uniformity of growth. For each of the three growth stages, rhizosphere soil was collected from each plant at three soil depths. The total sample size was therefore 54 (3 stages × 6 plants × 3 depths). Each composite sample was divided into two parts: one part of the samples was immediately stored in liquid nitrogen at −80 °C for DNA extraction; the other part of the samples was air-dried indoors and used for soil physicochemical property determination after passing through 2- and 0.149-mm soil sieves.

#### 2.2.2 Determination of the soil physical and chemical properties

Soil temperature was measured *in situ* at each soil depth using a portable electronic soil thermometer (±0.1 °C accuracy; Model TP-101, Zhangzhou, China) at the time of rhizosphere soil collection. The thermometer probe was inserted to the respective depth, and the reading was recorded after the value stabilized. Measurements were taken in triplicate at each depth. Soil pH was measured using a pH meter (PHSJ-4F, LeiCi, China) at a water:soil ratio of 5:1, following the method of Bao (2000). Total carbon (C) and total nitrogen (N) contents were determined using an elemental analyzer (Vario MACRO cube, Elementar, Germany). Total phosphorus (P) was determined using the sodium hydroxide fusion—molybdenum antimony colorimetric method (Bao, 2000). Soluble carbon (C) was measured using a TOC analyzer (enviro TOC, Elementar, Germany). Nitrate nitrogen and ammonium nitrogen were determined using the nitrosalicylic acid method and indophenol blue colorimetry, respectively, as described by Sun et al. (2025). All analyses were performed in triplicate.

#### 2.2.3 High-throughput sequencing of rhizosphere bacteria

The sequencing analysis was conducted by LC-Bio Technology Company (Hangzhou, China). Total DNA was eluted and preserved at −80 °C until PCR was performed. The 16S rRNA primers (341F, 5′-CCTACGGGGNGGCWGCAG-3′; 805R, 5′-GACTACHVGGGTATCTAATCC-3′) were amplified for bacteria. The sequencing results were analyzed using QIIME 2 (QIIME 2–2021.11; based on the NovaSeq PE 250 platform). All data were demultiplexed and quality filtered from raw fastq files using QIIME 2 (QIIME 2–2021.11) based on the following criteria: (1) removal of exact barcode matches and reads containing ambiguous characters; (2) PANDASeq (Masella et al., 2012), usage of double-ended Illumina reads and fast-assembled double-ended reads with the most errors corrected; and (3) truncation of reads at any site with a received mean quality score <20 using PRINSEQ (Pan et al., 2025). Furthermore, truncated reads with an N length of 5% of the total sequence length were discarded. Unassembled reads were discarded. QIIME 2 and DADA2 methods were used for primer removal, quality filtering, and denoising, with clustering at 100% similarity (i.e., into amplicon sequence variants, ASVs). To normalize sequencing depth (rarefaction), a custom Perl script was used to randomly subsample each sample to the minimum sequence count observed across all samples. For bacteria, the total raw sequences across 54 samples were 4,729,320; after rarefaction, each sample contained 3,415,122 sequences. For fungi, the total raw sequences were 4,370,790; after rarefaction, each sample contained 3,344,436 sequences. Rarefaction is critical for unbiased downstream comparisons, as microbial community composition varies with soil depth and growth stage. The sequences with the highest abundance in each amplicon sequence variant (ASV) were selected as representative sequences using QIIME 2 software. Default parameters were used in QIIME 2 software, and species annotation was performed using a pre-trained NaiveBayes classifier.

### 2.3 Data processing and analysis

In this study, SPSS 22.0 was used for statistical analysis, and Origin 2021 was used for graphing. First, the Shapiro–Wilk test was employed to verify the normal distribution of the data. Subsequently, one-way analysis of variance (ANOVA) was performed on the normally distributed data to determine the presence of significant differences, while the Kruskal-Wallis test and Mani test were applied to the non-normally distributed data. A Tukey HSD *post hoc* test was conducted for multiple comparisons. R (V3.6.2) was used to conduct rank sum tests for the *α*-diversity indices including Chao 1, Shannon, Simpson. The molecular ecological network analysis method based on random matrix theory (RMT) was adopted to analyze network parameters (such as average degree, average clustering coefficient, and average path length). Soil microbial communities at different soil depths and seasons were analyzed under default settings. Briefly, if certain species were not detected in half of the samples, they were removed, and the Pearson correlation coefficient was used to calculate the similarity matrix. To quantify the significant effects of different treatments on the overall structure (beta-diversity) of bacterial communities, permutational multivariate analysis of variance (PERMANOVA) was employed. Based on the Bray–Curtis dissimilarity matrix, a two-factor PERMANOVA was performed using the adonis2 function in the vegan package (version 2.6–4) to test the main effects and interaction of growth period (EP, MP, LP) and soil depth (0–20 cm, 20–30 cm, 30–40 cm). Statistical significance was assessed using 999 permutations. If a main effect was significant, post-hoc pairwise PERMANOVA tests were conducted, with *p*-values adjusted using the False Discovery Rate method. The Pearson correlation method was used to construct intra- or inter-domain co-occurrence networks of the soil microbial community. Topological parameters of the networks were derived from MENA, and visualization (with corresponding phylum levels added) was performed using Gephi (0.9.2). Based on correlation analysis and Mantel test, R (4.3.1) was used to analyze the correlations between soil environmental factors and microbial communities.

## 3 Results

### 3.1 Spatiotemporal heterogeneity of physicochemical properties in rhizosphere soil of *Atriplex canescens*

Taking the rhizosphere soil of *Atriplex canescens* as the research object, this study analyzed the variation characteristics of soil pH, soil temperature, total soil salt content, soil water content during the Early Growth Period (EP), Middle Growth Period (MP), Late Growth Period (LP), and at soil depths of 0–20 cm, 20–30 cm, and 30–40 cm. Nutrient contents in the rhizosphere soil of *A. canescens* among different soil depths (0–20 cm, 20–30 cm, 30–40 cm) and growth periods (EP, MP, LP) are shown in Table 1. Soil pH was generally distributed in a weakly alkaline range, with values ranging from 7.13 ± 0.04 to 7.70 ± 0.29. In the vertical dimension, the vertical stratification characteristics of pH varied among different growth periods: during EP, it showed an obvious “higher in deeper layers” trend, with the pH of the 30–40 cm layer (7.70 ± 0.29) being significantly higher than that of the 0–20 cm layer (7.38 ± 0.07; *p* < 0.05), while the 20–30 cm layer (7.52 ± 0.29) was in a transitional state with no significant difference from the upper and lower layers; in contrast, the pH showed strong vertical uniformity during MP and LP, with no significant differences detected among soil layers. From the perspective of the growth season timeline, the pH of all soil layers showed a consistent pattern—lowest in the mid-term: the pH of the 0–20 cm layer during MP (7.13 ± 0.04) was significantly lower than that during EP and LP (*p* < 0.05), and the 20–30 cm and 30–40 cm layers also showed similar mid-term acidification characteristics.

**Table 1:** Soil physicochemical properties of *Atriplex canescens* forest at different soil depths and growth periods.

Soil physicochemical properties	0–20 cm (EP)	0–20 cm (MP)	0–20 cm (LP)	20–30 cm (EP)	20–30 cm (MP)	20–30 cm (LP)	30–40 cm (EP)	30–40 cm (MP)	30–40 cm (LP)
pH	7.38 ± 0.07Ba	7.13 ± 0.04Ab	7.35 ± 0.17Aa	7.52 ± 0.29ABa	7.19 ± 0.09Ab	7.39 ± 0.22Aab	7.7 ± 0.29Aa	7.17 ± 0.06Ab	7.33 ± 0.09Ab
Soil temperature (°C)	19.82 ± 0.38Aa	32.45 ± 0.83Ab	9.27 ± 1.49Cc	18.53 ± 0.23Ba	30.83 ± 1.04ACb	10.18 ± 1.39Cc	17.27 ± 0.05Ca	30.07 ± 1.05BCb	11.37 ± 1.17Cc
Soil total salt content (g/kg)	9.97 ± 6.41Aa	4.33 ± 0.3Aa	11.58 ± 2.57Aa	8.28 ± 1.25Aa	4.29 ± 0.25Ab	8.96 ± 1.61Aac	6.16 ± 3.07Aa	4.28 ± 0.31Aa	7.14 ± 0.53Ba
Soil water content (%)	1.81 ± 0.17Aa	2.08 ± 0.51Aa	3.14 ± 1.21Ab	2.71 ± 0.43Ba	5.97 ± 2.33Bb	3.47 ± 1.46Aa	3.63 ± 0.64Ca	5.72 ± 0.03Bb	3.70 ± 1.37Aa
Total carbon content (g/kg)	13.07 ± 1.64Aab	11.6 ± 0.83Ab	13.69 ± 1.19Aa	12.49 ± 1.20Aab	11.66 ± 1.19Ab	13.69 ± 1.19Aa	11.21 ± 1.49Aa	10.54 ± 1.01Aab	9.63 ± 0.93Bb
Total nitrogen content (g/kg)	0.33 ± 0.02Aa	0.32 ± 0.01Aa	0.32 ± 0.02Aa	0.31 ± 0.01Aa	0.29 ± 0.01Bb	0.28 ± 0.01Bb	0.29 ± 0.02Ba	0.27 ± 0.01Cb	0.24 ± 0.01Cc
Total phosphorus content (g/kg)	0.56 ± 0.1Aa	0.62 ± 0.1Aa	0.61 ± 0.05Aa	0.55 ± 0.08Aa	0.63 ± 0.07Aa	0.55 ± 0.07ABa	0.43 ± 0.09Bb	0.57 ± 0.06Aa	0.49 ± 0.05Bab
Soluble carbon content (g/kg)	11.17 ± 0.61Aa	9.55 ± 0.47Ab	11.64 ± 0.86Aa	10.58 ± 0.59ABa	8.8 ± 0.47Bb	10.74 ± 1.10ABa	9.75 ± 0.84Ba	8.1 ± 0.56Cb	9.72 ± 1.01Ba
NO^3−^-N content (mg/kg)	47.23 ± 3.10Ab	53.33 ± 1.30Aa	54.57 ± 3.22Aa	41.41 ± 3.00Bb	49.07 ± 2.37Ba	48.35 ± 2.69Ba	37.91 ± 3.19Bb	44.28 ± 2.33Ca	43.42 ± 3.00Ca
NH^4+^-N content (mg/kg)	8.5 ± 0.32Bb	18.19 ± 2.28Aa	17.7 ± 0.76Aa	11.12 ± 1.62Ab	16.13 ± 2.23Aa	15.86 ± 0.90Ba	9.14 ± 2.05Bb	16.35 ± 1.94Aa	14.55 ± 0.65Ca

Different uppercase letters indicate significant differences between soil layers during the same growth period (*p* < 0.05), and different lowercase letters indicate significant differences between growth periods in the same soil layer (*p* < 0.05). The early growth period, middle growth period, late growth period are represented by EP, MP, and LP, respectively. Data are expressed as “mean ± standard deviation (SD)”.

Soil temperature displayed the most distinct spatiotemporal differences, with an overall fluctuating range between 9.27 ± 1.49 °C and 32.45 ± 0.83 °C. Vertically, all three growth periods showed a significant “surface enrichment” feature: during EP, the temperature of the 0–20 cm layer was significantly higher than that of the 20–30 cm and 30–40 cm layers, and the temperature decreased sequentially with the deepening of the soil layer; the vertical difference patterns during MP and LP were consistent with those during EP, only differing in numerical ranges. Temporally, the temperature of each soil layer synchronously showed a “mid-term peak” pattern: the temperature of the 30–40 cm layer during MP was significantly higher than that during EP and LP, and the 0–20 cm and 20–30 cm layers also had the highest temperature during MP, with significant differences from other periods (*p* < 0.05).

The total soil salt content was in the range of 4.28 ± 0.31 to 11.58 ± 2.57 g/kg. The vertical stratification effect only appeared in LP, where the total salt content of the 0–20 cm layer was significantly higher than that of the 30–40 cm layer (*p* < 0.05); in contrast, there were no significant differences in total salt content among soil layers during EP and MP. Temporally, the total salt content of each soil layer showed a common pattern of being lowest in the mid-term: the total salt content of the 0–20 cm layer during MP was lower than that during EP and LP, and the 20–30 cm and 30–40 cm layers showed the same numerical variation trend.

The soil water content was found to span a range of 1.81 ± 0.17% to 5.97 ± 2.33%, and its vertical distribution pattern changed significantly with the growth period. Both EP and LP showed a vertical characteristic of “higher in deeper layers”: during EP, the water content of the 30–40 cm layer was significantly higher than that of the 0–20 cm layer, and during LP, the 30–40 cm layer was also higher than the surface layer (*p* < 0.05); in contrast, MP showed a distribution pattern of “highest in the middle layer”, where the water content of the 20–30 cm layer was significantly higher than that of the 0–20 cm layer (*p* < 0.05). Temporally, the water content of each soil layer generally reached the highest in the middle growth period: the water content of the 20–30 cm layer during MP was significantly higher than that during EP and LP, and the 0–20 cm and 30–40 cm layers also had higher water content during MP than other periods (*p* < 0.05).

The total carbon (TC) content ranged from 9.63 ± 0.93 to 13.69 ± 1.19 g/kg, and the vertical differentiation showed a common “surface enrichment” feature, but significant differences were only observed in LP: the TC content of the 0–20 cm and 20–30 cm layers was significantly higher than that of the 30–40 cm layer (*p* < 0.05). Temporally, the TC content of each soil layer showed a pattern of “lower in the mid-term”, with higher values in EP and LP than in MP. Among them, the TC content of the 30–40 cm layer during LP was significantly lower than that during EP (*p* < 0.05).

The total nitrogen (TN) content was observed to fall within the range of 0.24 ± 0.01 to 0.33 ± 0.02 g/kg. Vertically, the TN content was highest in the surface layer (0–20 cm) during all growth periods: during EP, the TN content of the 0–20 cm layer was significantly higher than that of the 30–40 cm layer, and the difference between the surface layer and the deep layer was particularly significant during LP (*p* < 0.05). Temporally, the TN content showed a continuous decreasing trend with the progression of the growth period, which was most obvious in the 30–40 cm layer: the TN content during LP was significantly lower than that during EP and MP, and the 0–20 cm and 20–30 cm layers also showed a consistent decreasing trend (*p* < 0.05).

The total phosphorus (TP) content showed relatively mild overall spatiotemporal variation, with a fluctuation range of 0.43 ± 0.09 to 0.63 ± 0.07 g/kg. Vertically, significant differentiation was only observed in MP, where the TP content of the 30–40 cm layer was significantly higher than that during EP, and there were no significant differences in TP among soil layers during EP and LP (*p* < 0.05). Temporally, the TP content did not show a consistent pattern, and was only slightly higher in MP in some soil layers: the TP content of the 20–30 cm layer during MP was higher than that during EP, approaching the significance threshold, and there were no obvious differentiation characteristics among other soil layers.

The soluble carbon (SC) content ranged from 8.10 ± 0.56 to 11.64 ± 0.86 g/kg, showing a surface enrichment feature vertically, with the most significant soil layer differences in MP: the SC content of the 0–20 cm layer was significantly higher than that of the 30–40 cm layer, and during EP, the 0–20 cm layer was also significantly higher than the 30–40 cm layer (9.75 ± 0.84; *p* < 0.05). Temporally, the SC content showed a distinct “mid-term trough” feature, with the lowest SC content in all soil layers during MP. The SC content of the 20–30 cm layer during MP was significantly lower than that during EP and LP (*p* < 0.05).

The concentration of nitrate nitrogen (NO_3_^−^-N) varied between 37.91 ± 3.19 and 54.57 ± 3.22 mg/kg, and its vertical differentiation was significant throughout the growth season, showing a stable gradient distribution of “surface layer > middle layer > deep layer”. During EP, the NO_3_^−^-N content of the 0–20 cm layer was significantly higher than that of the 20–30 cm and 30–40 cm layers, and the vertical gradient differences during MP and LP were also significant (*p* < 0.05). Temporally, the NO_3_^−^-N content showed a steady increasing trend with the progression of the growth period: the NO_3_^−^-N content of the 0–20 cm layer during LP was significantly higher than that during EP, and the 20–30 cm and 30–40 cm layers also showed similar temporal increasing characteristics (*p* < 0.05).

NH_4_^+^-N concentrations exhibited a variation range of 8.50 ± 0.32 to 18.19 ± 2.28 mg/kg. Temporally, the NH_4_^+^-N content of each soil layer was significantly higher during MP and LP than during EP: the NH_4_^+^-N content of the 0–20 cm layer during MP was significantly higher than that during EP, and the inter-period differences of the 20–30 cm and 30–40 cm layers were also significant (*p* < 0.05). Vertically, there were no significant differences in NH_4_^+^-N among soil layers during all growth periods (*p* > 0.05).

The physicochemical properties of the rhizosphere soil of *A. canescens* showed significant spatiotemporal differentiation among different growth periods (EP, MP, LP) and soil depths (0–20 cm, 20–30 cm, 30–40 cm): temporally, during the middle growth period (MP), soil temperature, water content, and ammonium nitrogen peaked synchronously, while total salt and soluble carbon contents dropped to the lowest. Nitrate nitrogen increased with the growth process, and total nitrogen showed a decreasing trend; spatially, the surface soil had “surface enrichment” characteristics in pH (during EP), carbon and nitrogen nutrients, and water content (during EP and LP). During MP, the water content was “highest in the middle layer”; among all indicators, soil temperature and nitrate nitrogen showed the most significant differentiation, total phosphorus fluctuated gently.

### 3.2 *α*-Diversity of rhizosphere bacteria of *Atriplex canescens* among different soil depths and growth periods

**Figure 2:**
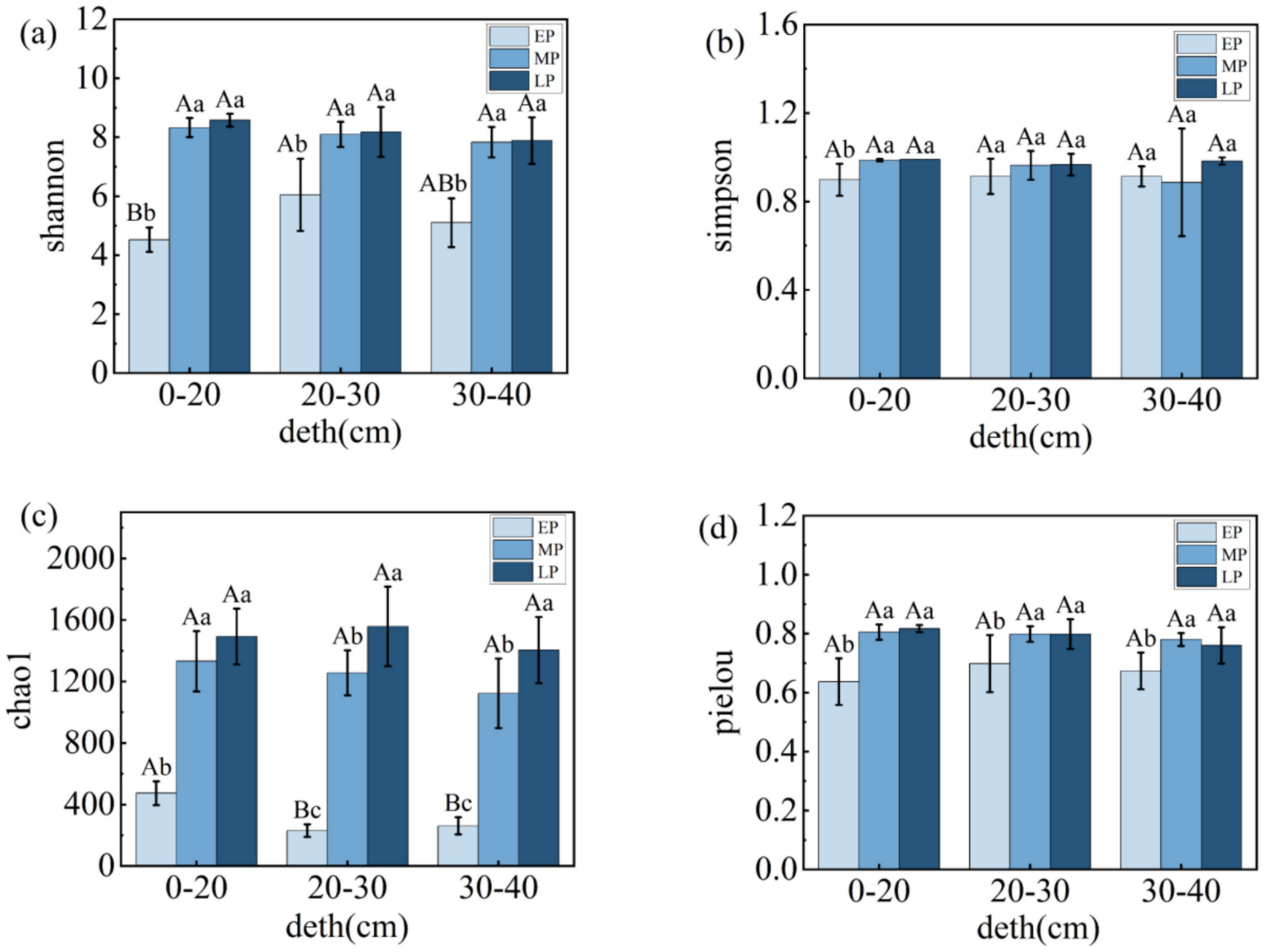
Rhizosphere soil bacterial community *α*-diversity. **(a)** Shannon, **(b)** Simpson, **(c)** Chao1, and **(d)** Pielou. EP, MP, and LP represent the early growth, middle growth, and late growth period, respectively. Different capital letters indicate significant differences (*p* < 0.05) among different soil layers during the same growth period, and different lowercase letters indicate significant differences (*p* < 0.05) among different growth periodsin the same soil layer.

The *α*-diversity of rhizosphere bacterial communities in the same soil layer differed significantly with growth period (*p* < 0.05, Figure 2). The Shannon, Simpson and Chao 1, indices of the rhizosphere bacterial communities in the 0–20 cm soil layer all showed that LP and MP were significantly higher than EP (*p* < 0.05), but there was no significant difference between MP and LP (Figures 2a–d). The rhizosphere bacterial communities in the 20–30 and 30–40 cm soil layers showed significant (*p* < 0.05) differences in the Shannon and Chao 1 indices at EP, MP, and LP, whereas the Simpson index showed no significant differences at EP, MP, or LP (Figures 2a–d). At EP, with increasing soil depth, the Shannon index was significantly higher in the 20–30 cm soil layer than in the 0–20 cm layer (*p* < 0.05). The Chao 1 index was significantly higher in the 0–20 cm layer than in the 20–30 and 30–40 cm layers (*p* < 0.05), whereas among soil depths, the Shannon, Simpson and Chao 1, indices showed no significant differences at MP or LP (Figures 2a–d).

The results of the two-way ANOVA (Table 2) indicated that growth period had a highly significant main effect on the Shannon, Simpson, Chao1 indices (*p* < 0.001). Soil depth had a significant effect only on the Chao1 index (*p* = 0.019), with no significant effect on the other indices (*p* > 0.05). A significant interaction effect between growth period and soil depth was observed for the Shannon, and Chao1 indices (*p* < 0.001).

**Table 2:** Effects of growth period and soil depth on *α*-diversity indices and overall community structure of rhizosphere bacteria in *Atriplex canescens*.

Source of variation	Dependent variable (analysis)	Statistic	df	Statistic value	Variance explained (*R*^2^/partial *η*^2^)	*p*-value
Growth period	Shannon index (Two-way ANOVA)	*F*	2	156.94	0.868 (partial *η*^2^)	<0.001
	Simpson index (Two-way ANOVA)	*F*	2	15.91	0.387 (partial *η*^2^)	<0.001
	Chao1 index (Two-way ANOVA)	*F*	2	190.56	0.886 (partial *η*^2^)	<0.001
Growth period	Community Structure (PERMANOVA)	*Pseudo-F*	2	5.50	0.174	<0.001
Soil depth	Shannon index (Two-way ANOVA)	*F*	2	3.07	0.066 (partial *η*^2^)	0.055
	Simpson index (Two-way ANOVA)	*F*	2	1.52	0.057 (partial *η*^2^)	0.228
	Chao1 index (Two-way ANOVA)	*F*	2	4.28	0.089 (partial *η*^2^)	0.019
	Community Structure (PERMANOVA)	*Pseudo-F*	2	1.84	0.055	0.004
Interaction	Shannon index (Two-way ANOVA)	*F*	4	7.17	0.235 (partial *η*^2^)	<0.001
	Simpson index (Two-way ANOVA)	*F*	4	2.53	0.161 (partial *η*^2^)	0.052
	Chao1 index (Two-way ANOVA)	*F*	4	13.23	0.496 (partial *η*^2^)	<0.001
	Community Structure (PERMANOVA)	*Pseudo-F*	4	0.91	0.061	0.702

Effect size reported as partial *η*^2^ for Two-way ANOVA and as *R*^2^ for PERMANOVA (based on Bray-Curtis distance, 999 permutations).

In summary, the *α*-diversity of the *A. canescens* rhizosphere bacterial community was significantly influenced by both growth period and soil depth, with an interaction effect present for some indices. The statistical tests confirmed that growth stage was the primary factor affecting all *α*-diversity indices.

### 3.3 Composition of rhizobacterial bacterial communities of *Atriplex canescens* at different soil depths and growth periods

**Figure 3:**
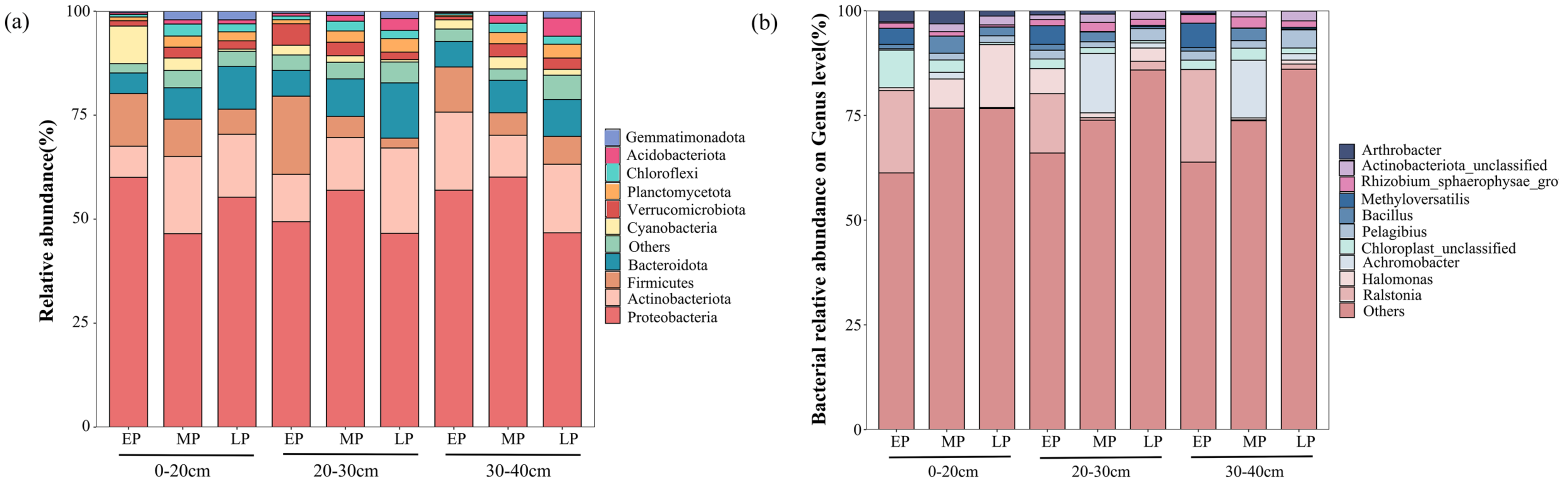
Relative abundance of bacteria at the phylum level **(a)** and genus level **(b)** in different soil layers across growth stages. EP, MP, and LP represent the early growth, middle growth, and late growth period, respectively.

PERMANOVA analysis revealed that the growth period had a highly significant impact on the overall community structure, explaining substantially more variation than soil depth (Table 2). The top 10 bacterial phyla in terms of relative abundance were selected for analysis, and the results are shown in Figure 3. At the phylum level, the dominant bacterial phyla were *Pseudomonadota*, *Actinomycetota*, *Bacillota*, *Bacteroidota*, *Cyanobacteria*, *Verrucomicrobiota*, *Planctomycetota*, *Acidobacteriota*, *Chloroflexota*, and *Gemmatimonadota*. In the 0–20 cm soil layer, the relative abundance of *Pseudomonadota* was significantly higher in EP than in MP and LP (*p* < 0.05), and the relative abundance of *Actinomycetota* increased significantly with the growth period (p < 0.05). In the 20–30 and 30–40 cm layers, the relative abundance of *Actinomycetota* first increased and then decreased. At the same soil depth, the relative abundance of *Bacillota* showed a decreasing trend with the growth period. During the EP period, the relative abundance of anamorphic mycorrhizal fungi gradually increased with the increase in the soil depth. Regardless of the soil depth, the relative abundance of thick-walled phyla was higher during EP than during MP and LP.

At the genus level, the top 10 bacterial genera in terms of relative abundance are shown in Figure 3b: *Ralstonia*, *Halomonas*, *Achromobacter*, Chloroplast_unclassified, *Pelagibius*, *Bacillus*, *Methyloversatilis*, *Rhizobium_sphaerophysae_*group, *Actinomycetota*_unclassified, and *Arthrobacter*. Within the 0–20 cm soil depth, the relative abundance of *Halomonas* spp. was shown to follow the order LP > MP > EP. Under the same soil layer conditions, the relative abundance of *Ralstonia* was significantly higher (*p* < 0.05) during EP than during MP and LP. The relative abundance of *Achromobacter* was significantly higher (*p* < 0.05) during MP than during EP in the 20–30 and 30–40 layers.

**Figure 4:**
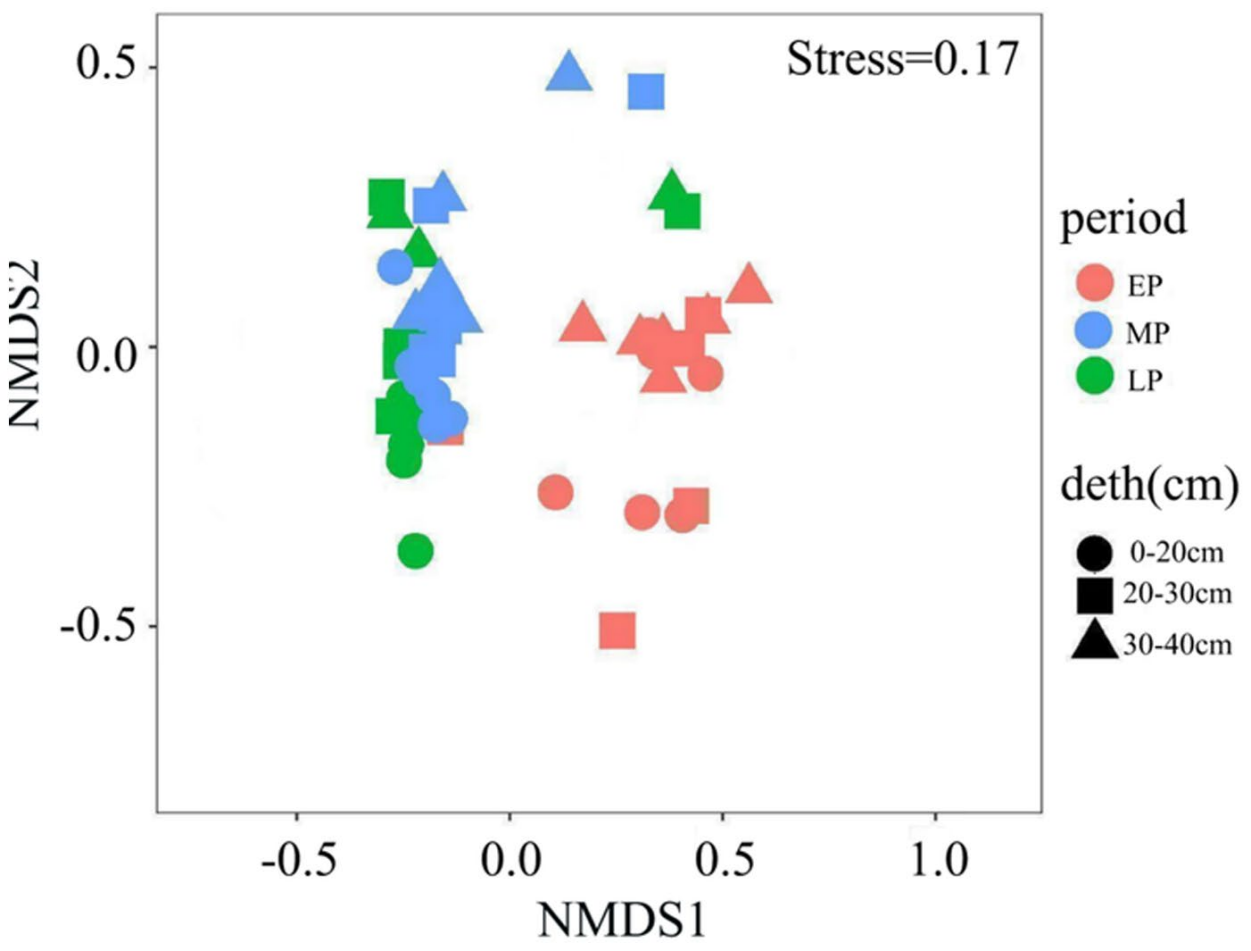
NMDS ranking of the soil bacterial community composition between roots at different soil depths and growth periods based on the Bray–Curtis distance. Note: EP, MP, and LP represent the early growth, middle growth, and late growth period, respectively.

Non-metric multidimensional scaling (NMDS) revealed the phase dissimilarity of rhizosphere bacterial communities at different soil depths during different growth periods (Figure 4). Stress was 0.17 (Stress < 0.2) under NMDS sorting, indicating the reliability of the analysis. EP was significantly separated from MP and LP under different growth period conditions, indicating that the species composition during EP significantly differed from that of MP and LP (*p* < 0.05).

In summary, both the growth period and soil depth significantly shaped the community structure of rhizosphere bacteria in *A. canescens*. At both the phylum and genus levels, the relative abundances of multiple key taxa exhibited specific variation patterns associated with either the growth period or soil depth. NMDS analysis further confirmed significant differences in bacterial community composition across different growth periods, with the EP period showing particularly distinct characteristics.

### 3.4 Effects of soil depths and growth periods on the co-occurrence network patterns of rhizosphere bacterial communities of *Atriplex canescens*

**Figure 5:**
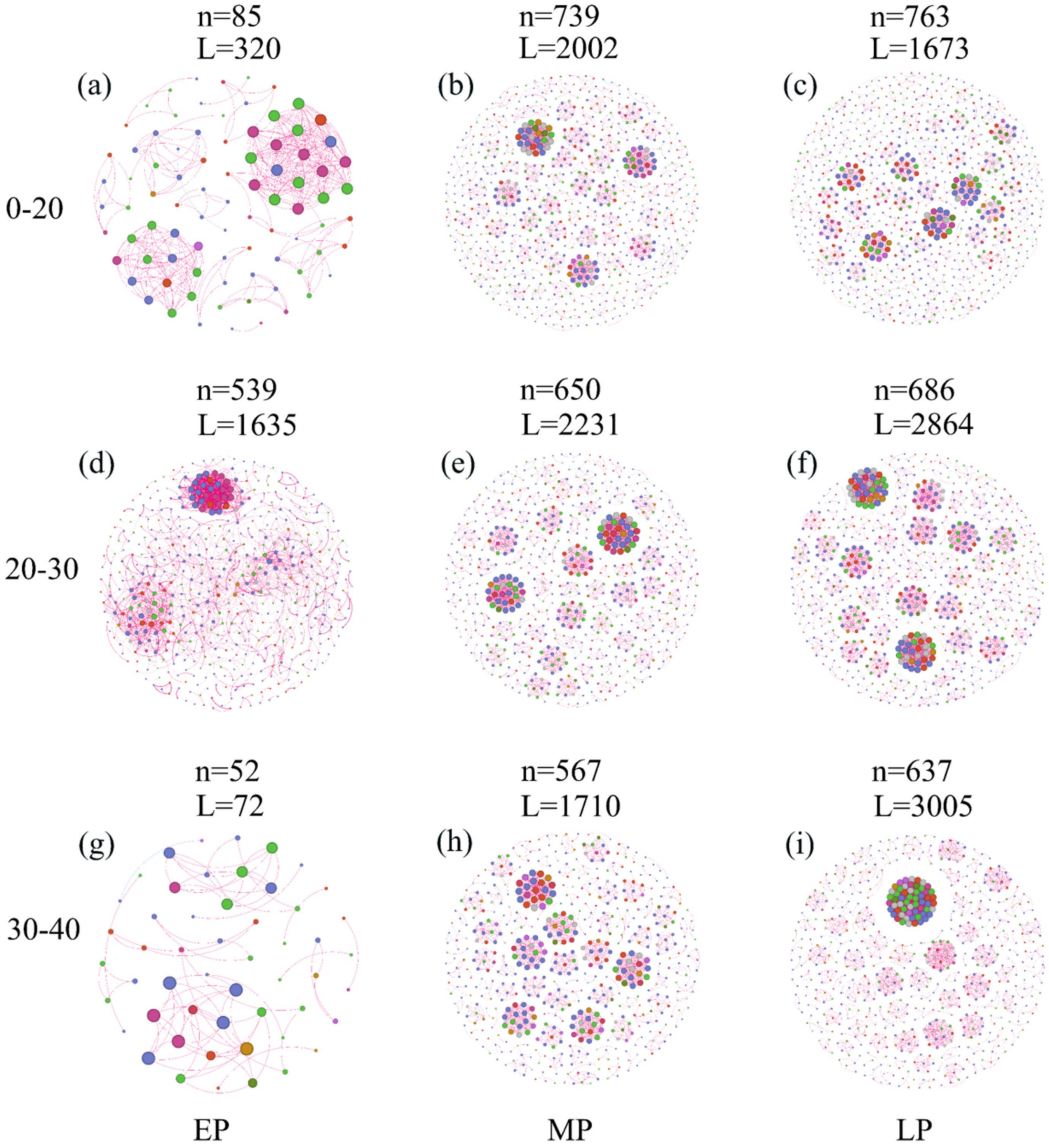
Rhizosphere bacterial co-occurrence network of four-winged shore quinoa at different soil depths during different growth periods. **(a–c)** 0–20 cm depth: **(a)** Early growth period (EP), **(b)** middle growth period (MP), **(c)** late growth period (LP); **(d–f)** 20–30 cm depth: **(d)** EP, **(e)** MP, **(f)** LP; **(g–i)** 30–40 cm depth: **(g)** EP, **(h)** MP, **(i)** LP. Different colors of nodes represent different bacterial phyla. Red lines represent positive correlations, and blue lines represent negative correlations. The node size represents the degree of centrality.

Co-occurrence networks based on Spearman’s correlation were constructed at the Amplicon Sequence Variant (ASV) level to explore the effects of soil depths and growth stages on the structure and interaction patterns of rhizosphere bacterial communities of *A. canescens* (Figure 5, Table 3). The results showed that the co-occurrence networks of bacterial communities under different treatments were dominated by positive correlations, accounting for 93.96% (0–20 cm, LP) to 99.82% (20–30 cm, EP).

**Table 3:** Topological indices of the co-occurrence network of rhizosphere bacterial communities during different growth periods.

Network parameters	0–20 cm			20–30 cm			30–40 cm		
	EP	MP	LP	EP	MP	LP	EP	MP	LP
Nodes	85	739	763	539	650	686	52	567	637
Edges	320	2002	1,673	1,635	2,231	2,864	72	1710	3,005
Number of positive connections	319/99.69%	1954/97.60%	1572/93.96%	1632/99.82%	2224/99.69%	2858/99.79%	71/98.61%	1692/98.95%	2992/99.57%
Number of negative connections	1/0.31%	48/2.4%	101/6.04%	3/0.18%	7/0.31%	6/0.21%	1/1.39%	18/1.05%	13/0.43%
Average degree	7.529	5.418	4.385	6.067	6.865	8.350	2.769	6.032	9.434
Network density	0.090	0.007	0.006	0.011	0.011	0.012	0.054	0.010	0.015
Network diameter	1	1	1	17.608	1	1	1	1	1
Modularity	0.702	0.948	0.967	0.743	0.927	0.932	0.840	0.951	0.711
Cluster	1	1	1	0.623	1	1	1	1	1

EP, MP, and LP represent the early growth, middle growth, and late growth period, respectively.

In the same soil layer, the number of nodes and edges in the networks during the MP and LP periods were significantly higher than those during the EP period, and the modularity index was higher in the LP period. During the EP period, obvious soil layer differentiation was observed: the network in the 20–30 cm soil layer (539 nodes, 1,635 edges) had a higher modularity index than those in the 0–20 cm and 30–40 cm soil layers.

Topological indices further revealed that the number of nodes and edges reached the maximum in the 30–40 cm soil layer during the LP period (637 nodes, 3,005 edges) and the minimum in all soil layers during the EP period (52–85 nodes, 72–320 edges). The average degree was the highest in the 30–40 cm soil layer during the LP period (9.434) and the lowest in all soil layers during the EP period (2.769–7.529).

The modularity index was higher than 0.7 in all treatments except the 30–40 cm soil layer during the LP period, with the highest value in the 0–20 cm soil layer during the LP period (0.967). The clustering coefficient was 1 for most treatments, and the network diameter was mainly 1. Only the 20–30 cm soil layer during the EP period were the values of clustering coefficient (0.623) and network diameter (17.608) distinct.

The parameters of rhizosphere bacterial co-occurrence networks showed significant differences under different growth periods and soil depth treatments. Positive correlations dominated in all networks. Network complexity (number of nodes, number of edges, average degree) was generally higher during the MP and LP compared to the EP. The modularity index was higher during the LP, while the network in the 20–30 cm soil layer during the EP period exhibited unique topological characteristics.

### 3.5 Mantel correlation analysis between dominant bacterial phyla and soil physicochemical factors

A Mantel test was conducted between the top four dominant bacterial phyla in terms of relative abundance and soil factors, revealing significant spatiotemporal heterogeneity in the key environmental factors influencing the rhizosphere bacterial community.

**Figure 6:**
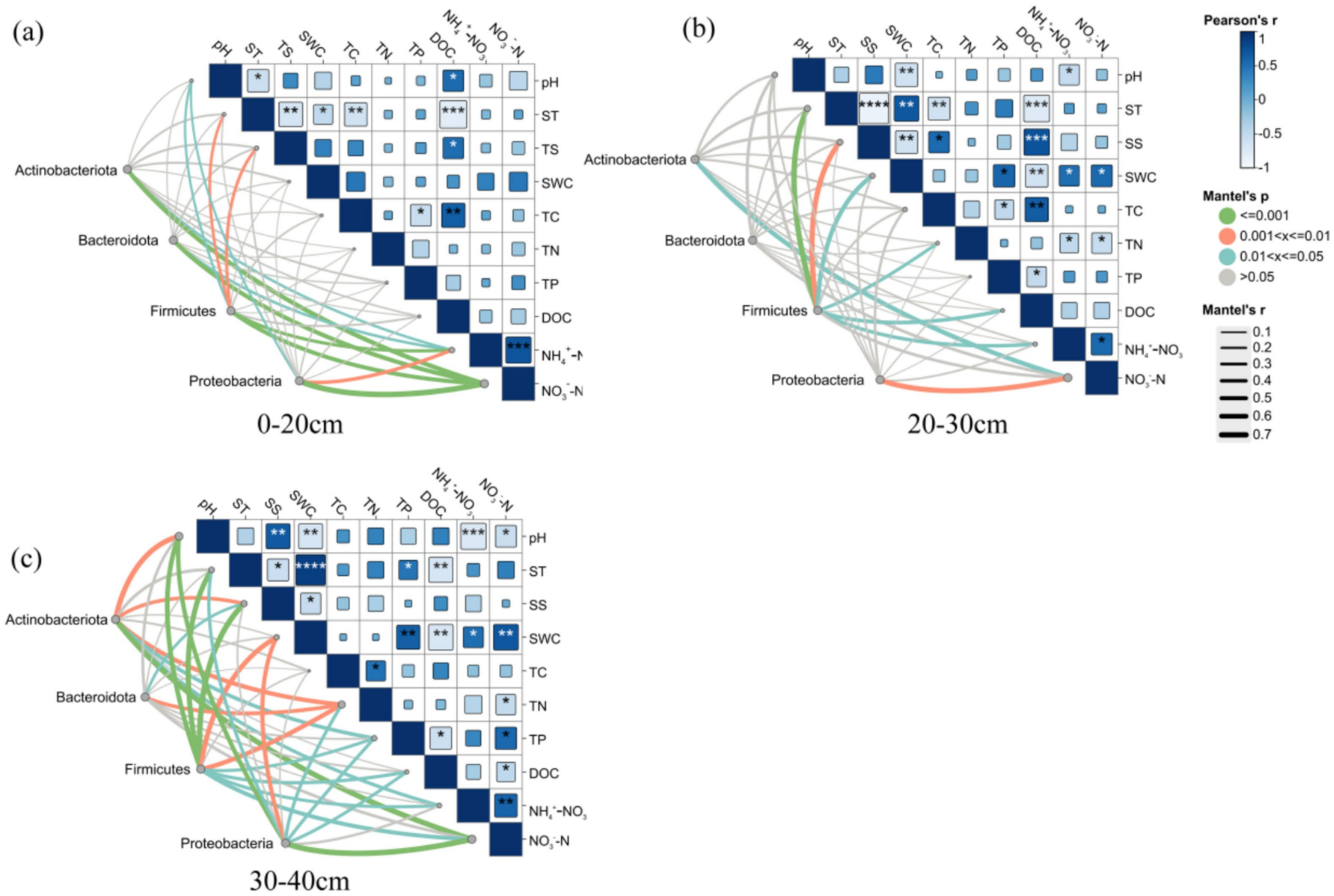
Mantel test results between rhizosphere bacterial community and soil properties in the **(a)** 0–20, **(b)** 20–30, and **(c)** 30–40 cm soil layers during different growth periods. EP, MP, and LP represent the early growth, middle growth, and late growth period, respectively. DOC represents dissolved organic carbon, TP represents total phosphorus, TN represents total nitrogen, and TC represents total carbon, ST represents soil temperature, TS represents total soil salt, SWC represents soil water content.

Firstly, from the perspective of the soil vertical profile, the number of soil factors significantly correlated with the dominant bacterial phyla increased with soil depth, demonstrating clear variation across soil depths (Figure 6). In the 0–20 cm surface soil layer, the bacterial community showed significant positive correlations primarily with NO_3_^−^-N, NH_4_^+^-N, soil temperature, and total soil salt (Figure 6a). In the 20–30 cm middle layer, the associated factors expanded to include more indicators such as DOC, TN, and soil water content (Figure 6b). In the 30–40 cm deep soil layer, in addition to the aforementioned factors, the TP and pH also exhibited significant positive correlations with the bacterial community (Figure 6c).

**Figure 7:**
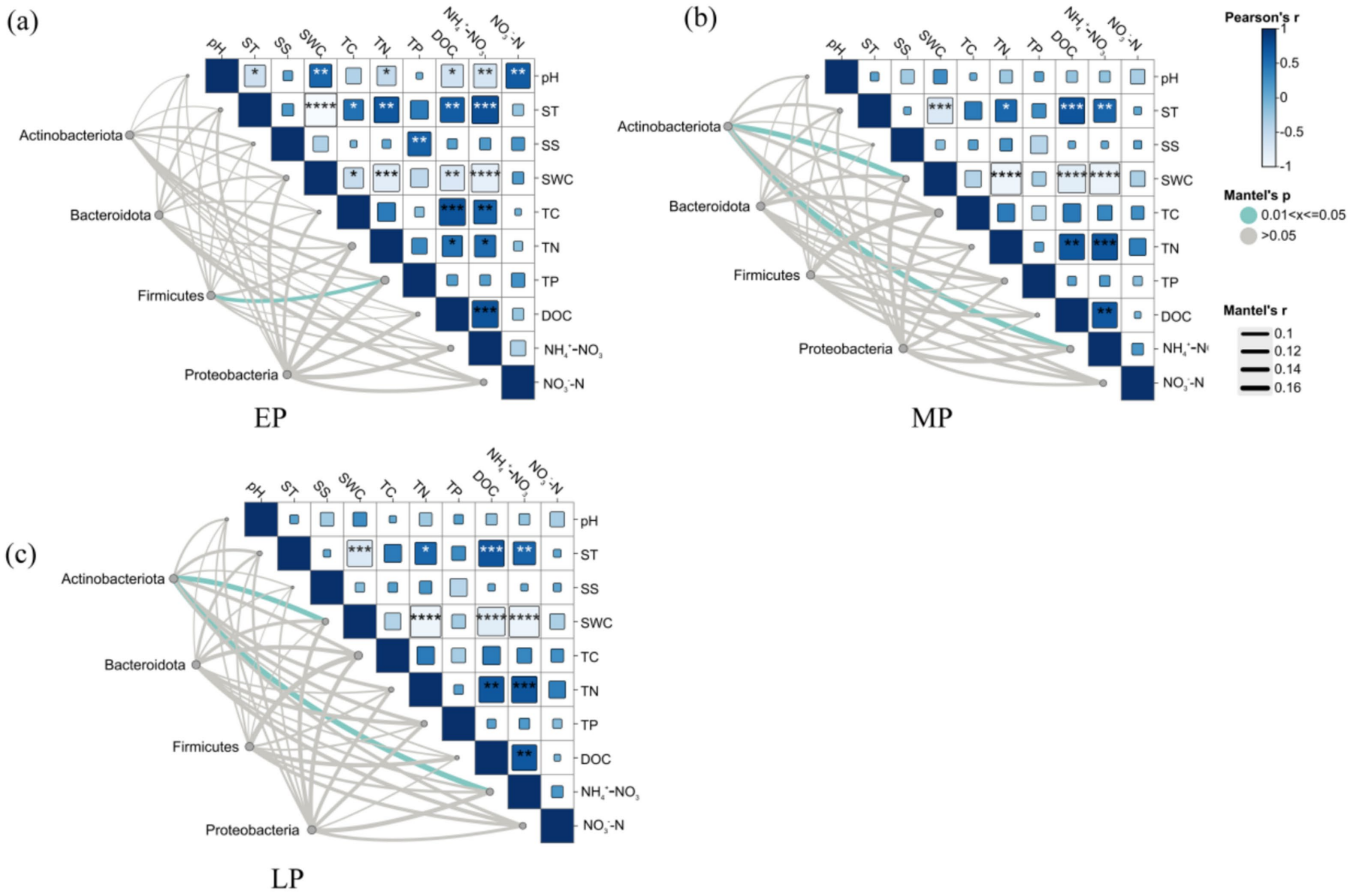
Mantel test results between rhizosphere bacterial community and soil properties in the different soil layers during the **(a)** EP, **(b)** MP, and **(c)** LP. EP, MP, and LP represent the early growth, middle growth, and late growth period, respectively. DOC represents dissolved organic carbon, TP represents total phosphorus, TN represents total nitrogen, and TC represents total carbon, ST represents soil temperature, TS represents total soil salt, SWC represents.

Secondly, in terms of seasonal growth dynamics, the key driving factors displayed distinct variation across growth period (Figure 7). During the EP, the dominant bacterial phyla showed a significant positive correlation only with TP content (Figure 7a). As the growth progressed into the MP and LP, NH_4_^+^-N and soil water content became the predominant common factors positively correlated with the community (Figures 7b,c).

In summary, the Mantel test results indicate that the soil factors significantly associated with the dominant bacterial phyla exhibit differences in both vertical distribution and seasonal dynamics. The number and types of significantly correlated factors increase and expand with soil depth, while the dominant associated factors differ across various growth periods.

Mantel tests revealed that the soil factors significantly correlated with rhizosphere bacterial community structure varied across different soil layers, and the number of significantly correlated factors increased with soil depth.

## 4 Discussion

The vulnerability and restoration difficulty of desert ecosystems in extreme arid regions determine that the synergistic interaction between plants, rhizosphere microorganisms, and soil is the core link for vegetation colonization, survival, and stable ecological functions (Schulz-Bohm et al., 2018). As a dominant shrub for desert restoration in this region, the spatiotemporal dynamics of rhizosphere bacterial communities in *A. canescens* not only reflect the adaptive strategies of microorganisms to extreme environments but also reveal the adaptation mechanism between plants and microecosystems.

### 4.1 Spatiotemporal differentiation characteristics of rhizosphere bacterial communities

Both the composition and structure of rhizosphere bacterial communities in *A. canescens* show significant spatiotemporal differentiation, which is highly coupled with plant growth rhythm and soil layer microenvironmental heterogeneity. To clearly separate the two driving factors, the following subsections first describe the effects of growth stage and then the effects of soil depth.

#### 4.1.1 Compositional dynamics: contrasting patterns between growth stage and soil depth

At the phylum level, *Pseudomonadota*, *Actinomycetota*, *Bacillota*, and *Bacteroidota* are the dominant taxa in all treatment groups, accounting for over 70% of the total relative abundance. This result is consistent with the findings of Li et al. (2024a) in the study of rhizosphere microorganisms of desert plant *Hippophae rhamnoides* and Zhang et al. (2012) in the investigation of rhizosphere bacterial communities of *Haloxylon ammodendron*, confirming that these phyla are core functional groups involved in material cycling, energy flow, and stress adaptation in desert ecosystems. Their spatiotemporal dynamics can serve as biological indicators of rhizosphere microenvironmental changes, reflecting the common pattern of rhizosphere bacterial community composition in desert plants.

From the perspective of the temporal dimension, the bacterial communities in the root-intensive layers (20–30 cm and 30–40 cm) are most sensitive to plant growth rhythm: the relative abundance of *Actinomycetota* in the MP and LP is significantly higher than that in the EP (*p* < 0.05), forming an evidence chain with the physicochemical characteristic of peak contents of soil ammonium nitrogen (NH₄^+^-N) and nitrate nitrogen (NO_3_^−^-N) in the same period. Due to its dual functions of complex organic matter degradation and plant growth promotion (Layeghtifard et al., 2017), the increased abundance of *Actinomycetota* can efficiently convert rhizosphere organic substrates into available nutrients, precisely meeting the nutrient demands of *A. canescens* during the vigorous growth stage. This characteristic of “growth period-dominated dynamic changes in functional bacterial phylum abundance” is consistent with the rule of “enrichment of growth-promoting bacterial phyla during the vigorous growth period” found in *Hippophae rhamnoides* by Li et al. (2024b), further confirming that the directional selection of rhizosphere bacterial communities by plant growth rhythm is a common adaptive strategy of desert plants.

At the genus level, the dynamic changes of *Ralstonia*, a plant pathogenic bacterium, further strengthen this rule: the relative abundance of this genus is significantly enriched during the EP period (reaching 8.3% in the 20–30 cm soil layer), showing a strong correlation with the significantly lower *α*-diversity of the community during MP and LP (*r* = −0.68, *p* < 0.01). This is consistent with the conclusion of Liu H. et al. (2022) in the study of tomato bacterial wilt that “invasion of *Ralstonia* can reduce the diversity of rhizosphere bacterial communities through competitive inhibition or metabolic toxicity”, indicating that the impact of dynamic changes of harmful pathogenic bacterial communities on community structure is universal across plant species. With the growth of *A. canescens*, root development is gradually improved, and the directional selection effect on rhizosphere bacterial communities is enhanced. The abundance of *Ralstonia* continues to decrease (only 1.2% in the 20–30 cm soil layer during LP), while bacterial genera such as *Halomonas* and *Arthrobacter*—which have been reported as plant growth-promoting rhizobacteria (PGPR) with salt tolerance and growth-promoting traits (Zhang X. et al., 2024; AlQuwaie et al., 2025; Li et al., 2025)—are gradually enriched. A temporal evolutionary pathway is formed with plant growth as the starting point, by inhibiting harmful bacterial communities and enriching functional bacterial communities, and ultimately achieving an increase in diversity. This explains the physiological phenomenon that *A. canescens* has faster growth rate and more biomass accumulation during MP and LP from the microbial perspective (Barrow et al., 2008).

Turning to the spatial dimension, the *α*-diversity and the abundance of functional groups of bacterial communities in the root-intensive layers (20–30 cm and 30–40 cm) are significantly higher than those in the topsoil (0–20 cm; *p* < 0.05), showing the characteristic of “enrichment in root-intensive layers”. This is directly related to the microenvironmental advantages of the concentrated distribution of *A. canescens* roots: the root-intensive layers have high organic matter content and active nutrient cycling, providing sufficient conditions for the growth and reproduction of bacterial communities, which is consistent with the research results of most desert plants that “root distribution areas drive bacterial community enrichment” (Estrada et al., 2024). It is worth noting that a “suitable microzone” is formed in the 20–30 cm soil layer during the EP period, and the complexity of its co-occurrence network structure (539 nodes, 1,635 edges) is higher than that in the 30–40 cm and 0–20 cm soil layers. This characteristic stems from the relatively stable moisture conditions of this soil layer, avoiding the salt accumulation in the 0–20 cm topsoil and the water scarcity in the 30–40 cm deep soil, and becoming the core area for early bacterial interactions. This finding enriches the theoretical understanding of bacterial community differentiation in the root-intensive layers of desert plants and reflects the specific shaping effect of extreme arid environments on the spatial distribution of bacterial communities.

In summary, the spatiotemporal differentiation of community composition is highly adapted to the plant growth demands, directly providing support for Hypothesis 1: “Through the regulation of growth periods and adaptation to soil layer microenvironments, *A. canescens* drives significant spatiotemporal differences in rhizosphere bacterial communities that are adapted to growth demands”.

4.1.2 Structural responses: network complexity under temporal and spatial gradients.

As a core method to analyze microbial interspecific interaction patterns and community stability (Li et al., 2018), co-occurrence network analysis reveals that the spatiotemporal differentiation of community structure is a functional extension of compositional differentiation. Regardless of different soil depths or growth periods, the interspecific associations of rhizosphere bacterial communities in *A. canescens* are dominated by positive correlations, with the proportion of positive correlation connections ranging from 93.96% (30–40 cm, LP) to 99.82% (20–30 cm, EP). This characteristic is consistent with the network analysis results of *Haloxylon ammodendron* rhizosphere by Zhang S. et al. (2024) and *Hippophae rhamnoides* rhizosphere by Li et al. (2024a,b) and is a common strategy for rhizosphere microecosystems to adapt to extreme arid environments (Layeghtifard et al., 2017): positive correlations mainly correspond to interspecific synergistic interactions, including symbiotic nitrogen fixation, cross-feeding metabolism, and secretion of stress-resistant substances. These can effectively reduce the impact of environmental fluctuations on individual species, significantly improve the overall resistance and resilience of the community, and provide stable microecological support for plant growth under stress.

First, considering the temporal dimension, the number of nodes and edges in the co-occurrence network during MP and LP is significantly higher than that during EP, and the modularity index is higher (reaching 0.967 in the 20–30 cm soil layer during LP). According to the network stability theory proposed by Krause et al. (2003), the higher the modularity, the more distinct the community functional partitioning and the stronger the anti-interference ability. This result is consistent with the higher *α*-diversity of the community during MP and LP, and also consistent with the rule of “more stable network structure during the vigorous growth period” found in *Hippophae rhamnoides* by Li et al. (2024a,b), indicating that the bacterial communities during the vigorous growth stage have formed a “modular structure” with functional specialization. Each sub-module can focus on specific ecological functions, which not only reduces resource competition and interference between different functional groups but also improves the efficiency of functional execution through close collaboration within the module, perfectly adapting to the high demand for rhizosphere ecological services by plants. In contrast, although the network connection density is high during the EP period, the modularity is low (only 0.702 in the 20–30 cm soil layer during EP). This characteristic reveals the adaptive strategy of bacterial communities to efficiently utilize limited resources through “high-density collaboration” when nutrients are insufficient in the early stage. The low modularity stems from the high niche overlap of most species in the community at this time, and no clear functional division of labor has been formed (Li et al., 2018). Similar results of this early adaptive strategy have been found in rhizosphere studies of other desert plants (Zhang et al., 2012).

Second, regarding the spatial dimension, the complexity and particularity of the network structure in the 20–30 cm soil layer further reflect the synergistic regulatory effect of soil layer microenvironment on bacterial community structure— this soil layer has relatively suitable moisture and salt conditions, becoming the “optimal microzone” for bacterial interactions, which is highly consistent with the “soil layer microenvironment adaptation shaping community structure” in Hypothesis 1. In summary, the spatiotemporal differentiation law of community structure echoes the compositional differentiation, jointly confirming the rationality of Hypothesis 1, that is, *A. canescens* drives significant spatiotemporal differences in rhizosphere bacterial community structure through the synergistic regulation of growth periods and soil layer microenvironments, and these differences are highly adapted to plant growth demands.

### 4.2 Core regulatory chain of spatiotemporal differentiation

#### 4.2.1 Dominant regulatory role of plant growth periods

This subsection focuses solely on the regulatory effect of plant growth stage, independent of soil depth. Plant growth rhythm is the core factor driving the spatiotemporal differentiation of communities, and its regulatory pathway is mainly mediated through the root development process: during the EP period, the distribution range of *A. canescens* roots in the 20–40 cm intensive layer is limited, and the development is not yet complete. The modification effect on the rhizosphere microenvironment is weak, and the directional selection effect on bacterial communities is not significant, leading to the enrichment of taxa with strong stress resistance but single function (such as *Pseudomonadota*, *Ralstonia*), resulting in low community diversity and simple structure. After entering the MP and LP periods, the plants grow vigorously or enter the reproductive growth stage, the root biomass in the 20–40 cm root-intensive layer increases significantly, the modification effect on the rhizosphere microenvironment is enhanced, providing more suitable living conditions for bacterial communities, promoting the enrichment of functional bacterial communities, and inhibiting the growth and reproduction of harmful bacterial communities (such as *Ralstonia*; Liang et al., 2019). Finally, the community diversity is improved and the structure tends to be stable.

If the low community diversity during the EP period is solely caused by extreme drought stress, the diversity of all soil layers should be significantly lower than that during MP/LP. However, the actual results show that the diversity of the 20–30 cm soil layer during EP (Shannon = 3.21) is higher than that of the 0–20 cm (2.97) and 30–40 cm (3.05) soil layers. And this soil layer not only maintained relatively higher moisture and total nitrogen content during the EP, but its soil temperature and pH also fell within a milder and more stable range, while the total salt content was comparatively lower. This indicates that, despite the overall drought stress during the EP, the 20–30 cm soil layer provided a more favorable micro-environment in terms of multiple environmental conditions such as moisture, temperature, pH, and salinity. Therefore, environmental stress primarily sets the overall lower baseline for community diversity, whereas the differential shaping of micro-environments across soil layers—mediated by plant growth rhythm—serves as the core driving factor behind the divergence in diversity among different soil layers. This indicates that environmental stress is an auxiliary regulatory factor, and the microenvironmental changes mediated by plant growth rhythm are the core driving factor leading to differentiation. This conclusion is consistent with the research results on the regulatory mechanism of rhizosphere bacterial communities in most desert plants (Liang et al., 2019). The clarification of this dominant regulatory role further confirms the core logic of “growth period regulation driving community differentiation” in Hypothesis 1.

#### 4.2.2 Synergistic regulatory role of soil depth

This subsection focuses solely on the regulatory effect of soil depth, independent of growth stage. Soil depth drives community differentiation synergistically with plant growth periods by shaping microenvironmental heterogeneity. Due to the dense root distribution and accumulation of litter decomposition products, the 20–30 cm and 30–40 cm root-intensive layers form a “high-nutrient, high-activity” microenvironment, where the bacterial communities are dominated by functionally diverse taxa with the highest diversity and metabolic activity. The 0–20 cm topsoil has sparse root distribution, insufficient organic matter input, and is vulnerable to extreme environmental stress (such as salt accumulation, moisture fluctuation), so the bacterial communities are dominated by stress-tolerant taxa (such as *Bacillota*), with significantly reduced diversity and functional complexity. This vertical differentiation characteristic is highly consistent with the vertical distribution law of soil physicochemical properties, and also consistent with the rule of “root distribution areas regulating the vertical differentiation of bacterial communities through resource distribution” found in the rhizosphere of *Haloxylon ammodendron* by Zhang et al. (2012), indicating that the synergistic regulatory role of soil depth on bacterial communities is universal.

The “suitable microzone” characteristic of the 20–30 cm soil layer during the EP period in this study is the product of the combination of the specific vertical distribution of soil moisture and salt in extreme arid regions and the regulatory role of the root-intensive layer—this soil layer not only avoids the strong stress of the topsoil but also makes up for the resource scarcity of the 30–40 cm deep soil, becoming the core area for bacterial interactions, reflecting the differentiated performance of common mechanisms in special environments.

#### 4.2.3 Mediating role of soil physicochemical properties

Soil physicochemical properties are the key intermediaries connecting plant regulation and bacterial community differentiation, and their spatiotemporal heterogeneity directly drives changes in community composition and structure. The analysis of this mechanism directly verifies Hypothesis 2. The results of the Mantel test show that there are significant differences in the dominant regulatory factors among different soil layers (*p* < 0.05): the bacterial communities in the 0–20 cm topsoil are significantly correlated with NO_3_^−^-N and pH (*r* = 0.48–0.53); the core area of the 20–30 cm root-intensive layer focuses on nitrogen (NO_3_^−^-N, NH₄^+^-N, TN) and dissolved organic carbon (DOC; *r* = 0.57–0.63); the 30–40 cm root-intensive layer is comprehensively regulated by nitrogen (NO_3_^−^-N, NH₄^+^-N, TN), phosphorus (TP), DOC, and pH (*r* = 0.45–0.61).

As a limiting element for bacterial growth and reproduction (Siles and Margesin, 2016), the spatiotemporal dynamics of nitrogen play a particularly critical regulatory role in the community: the increase in soil nitrogen content in the root-intensive layer during MP and LP provides the necessary material basis for the growth of dominant bacterial communities, directly driving the increase in the abundance of functional bacterial phyla such as *Actinomycetota*; the vertical distribution difference of nitrogen (root-intensive layer > topsoil) further exacerbates the soil layer differentiation of the community. This rule of “nitrogen-dominated bacterial community regulation” is consistent with the research results of most desert soil bacteria (Siles and Margesin, 2016), reflecting the core regulatory status of nitrogen in extremely impoverished environments. As a core physicochemical factor of desert soil, the significant correlation between pH and dominant bacterial phyla (*Pseudomonadota*, *Bacillota*) reveals a two-way interaction mechanism: such bacterial communities can regulate soil ion balance by secreting acidic metabolites (such as lactic acid, citric acid) or accumulating compatible solutes (such as betaine; Liang et al., 2024), indirectly reducing soil alkalinity. This not only improves the pH microenvironment for their own survival but also increases the solubility of nutrients such as nitrogen and phosphorus in the soil, creating favorable conditions for the absorption of nutrients by *A. canescens* roots, forming a synergistic interaction cycle of “plant-microorganism-soil.” This mechanism has also been confirmed in related studies on alkaline desert soils (Liang et al., 2024).

It is worth noting that with the increase of soil depth, the number of soil factors significantly correlated with the community increases, indicating that the bacterial communities in the 30–40 cm soil layer need to adapt to multiple stresses such as uneven nutrient distribution and high pH, and are more sensitive to changes in various physicochemical factors; while the bacterial communities in the 20–30 cm soil layer, due to the advantage of resource supply, show more concentrated responses to environmental factors on nitrogen and dissolved organic carbon. This rule is consistent with the research results on the environmental adaptability of microorganisms in the root-intensive layers of other arid region plants (Zhang et al., 2012), further confirming the universality and environmental specificity of the mediating role of soil physicochemical properties. The above results fully verify Hypothesis 2: “Soil physicochemical properties are the key factors mediating the spatiotemporal regulation of rhizosphere bacterial community structure by *A. canescens*, and there are differences in the dominant regulatory factors among different soil depths”.

In conclusion, this study forms the core regulatory chain of the spatiotemporal dynamics of rhizosphere bacterial communities in *A. canescens* in extreme arid regions: the synergistic effect of the growth period and soil depth of *A. canescens* drives the spatiotemporal heterogeneity of soil physicochemical factors such as nitrogen, dissolved organic carbon, and pH through the development process of the 20–40 cm root-intensive layer; this heterogeneity further specifically screens adaptive functional bacterial communities and inhibits harmful pathogenic bacterial communities, ultimately leading to significant spatiotemporal differentiation in community composition and structure, and this differentiation is highly adapted to the demands of *A. canescens* at different growth stages—during MP and LP, the stable and efficient bacterial community structure in the root-intensive layer provides ecological services such as nutrient transformation and stress resistance for vigorous plant growth; during EP, the bacterial community enrichment in the “suitable microzone” of the 20–30 cm soil layer ensures the successful colonization of plants in extreme environments. This chain completely connects the synergistic relationship of “plant–soil-microorganism,” not only confirming the common rules of rhizosphere bacterial community regulation in desert plants but also highlighting the specific findings brought by the subdivided sampling of root-intensive layers in extreme arid environments.

## 5 Conclusion

This study took *A. canescens* in extreme arid regions as the research object, adopting multi-dimensional technical methods including high-throughput sequencing, co-occurrence network analysis, and PERMANOVA. The main findings are as follows: the structure of the rhizobacterial community exhibited significant spatiotemporal differentiation dependent on both growth period and soil depth. Temporally, pathogenic-related genera were relatively enriched in the EP, whereas functional groups dominated during MP and LP. Spatially, the 20–40 cm soil layer, characterized by dense root distribution, served as a key zone for microbial enrichment. Specifically, during EP, the 20–30 cm layer, with its suitable moisture and salinity conditions, formed a “favorable micro-zone” for early microbial interactions. The observed community differentiation was jointly driven by plant growth phase and soil depth, mediated primarily through soil physicochemical properties—particularly nitrogen, available carbon, and pH. Growth phase governed microbial succession via root development, while soil depth filtered microbial composition through microenvironmental heterogeneity. Nitrogen and available carbon mainly regulated the root-dense layer, whereas pH exerted significant effects in both surface and deeper layers.

The innovation of this study lies in clarifying the spatiotemporal pattern of the rhizobacterial community of *A. canescens* and the synergistic “plant–soil–microbe” regulatory pathway, thereby revising the conventional view that “the surface layer is always the hotspot of microbial activity” and deepening the understanding of plant–microbe co-adaptation mechanisms in extremely arid zones. On a practical level, the findings provide a basis for precise regulation of the rhizosphere microecology in desert restoration: it is recommended to enhance nitrogen and available carbon supply in the root-dense layer during MP/LP, while optimizing moisture and salinity conditions in the 20–30 cm layer during EP, so as to promote the establishment of functional microbiota, suppress pathogenic groups, and ultimately improve the colonization efficiency of *A. canescens* and ecosystem stability.

## Data Availability

The raw data generated in this study can be found in Mendeley Data via doi: https://data.mendeley.com/datasets/xxhpknx75g/1.
